# The shared microbiome in mud crab (*Scylla paramamosain*) of Sanmen Bay, China: core gut microbiome

**DOI:** 10.3389/fmicb.2023.1243334

**Published:** 2023-09-01

**Authors:** Xiaosong Jiang, Mingming Niu, Kangxiang Qin, Yun Hu, Yuntao Li, Chenxi Che, Chunlin Wang, Changkao Mu, Huan Wang

**Affiliations:** ^1^School of Marine Science, Ningbo University, Ningbo, Zhejiang, China; ^2^Key Laboratory of Aquacultral Biotechnology, Ministry of Education, Ningbo University, Ningbo, Zhejiang, China

**Keywords:** *Scylla paramamosain*, core gut microbiota, microbial community stability, probiotic, Sanmen Bay

## Abstract

**Introduction:**

The mud crab, *Scylla paramamosain*, holds great commercial significance as a marine crustacean widely cultivated in the Indo-Pacific region. Understanding the core gut microbiota of aquatic animals is crucial for their overall health and growth, yet the core gut microbiota of mud crab remains poorly characterized.

**Methods:**

In this study, we gathered gut samples from mud crabs across five locations within Sanmen Bay, China. Through the utilization of high-throughput sequencing, we delved into the composition of the gut microbial community and identified the core gut microbiome of mud crab.

**Results:**

Our results demonstrate that the gut microbial diversity of mud crab did not exhibit significant variation among the five sampling sites, although there were some differences in community richness. At the phylum level, we identified 35 representative phyla, with Firmicutes, Proteobacteria, Bacteroidota, and Campilobacterota as the dominant phyla. Among the 815 representative genera, we discovered 19 core genera, which accounted for 65.45% of the total sequences. These core genera were distributed across 6 phyla, and among them, *Photobacterium* exhibited the highest average relative abundance.

**Discussion:**

*Photobacterium* has probiotic activity and may play a crucial role in enhancing the immune response of the host and maintaining the diversity of the gut microbiota. Moreover, we observed a positive correlation between the relative abundance of core genera and the stability of the gut microbial community. Furthermore, our findings revealed distinct differences in gut microbial composition and specific taxa between the sexes of mud crab. These differences subsequently influenced the functionality of the gut microbial community. Overall, our investigation sheds light on the core gut microbiota of mud crab, emphasizing the importance of core gut microbial communities in maintaining the health and growth of these commercially significant marine crustaceans.

## 1. Introduction

The mud crab, *Scylla paramamosain*, holds significant economic importance and has become a major contributor to the global seafood market (Tang et al., 2020; Perea and Festijo, 2022). Renowned for its rapid growth, large size, high meat yield, and delectable flavor, the mud crab is widely favored in the coastal regions of southern China. In 2022, China’s cultivation area for mud crabs reached an extensive 24,170 hectares, with an annual production of 154,661 tons. This production accounts for over half of the total marine aquaculture output in the country (Wang et al., 2019; Xie et al., 2021; China Fishery Statistical Yearbook, 2023).

Situated in the central region of Zhejiang Province, Sanmen Bay (29°02′53″N, 121°42′38″E) is a semi-enclosed bay that faces the East China Sea and falls under the jurisdiction of Sanmen County. With an approximate area of 775 square kilometers and an average water depth of 5–10 meters, the bay is renowned for its fishing and seawater aquaculture (Liu et al., 2018). Due to the favorable water quality in Sanmen Bay, which is ideal for mud crab cultivation, as well as its long-established history and efficient cultivation techniques, Sanmen Bay has emerged as one of the primary production areas for high-quality mud crab in China. The mud crabs from Sanmen have long enjoyed a reputation for their superior quality and are acknowledged as the finest specimens in the field. Sanmen County, often referred to as the “Hometown of Chinese mud crab” encompasses Huaqiao Town, Jiantiao Town, Pubagang Town, Shaliu Town and Shepan Town, where mud crabs are artificially bred in seawater ponds utilizing the bay’s resources. These regions serve as the primary breeding grounds for mud crabs in Sanmen Bay.

Research has demonstrated the influence of gut microbes on host growth and development, immunity, metabolism and nutrition (Rolfe, 1984; Sánchez et al., 2017). Gut microbes play a vital role in maintaining gut health by producing vitamins and enzymes that aid in nutrient digestion and absorption, replenishing vitamins and inhibiting the growth of harmful bacteria (Tremaroli and Bäckhed, 2012; Wei et al., 2019). The relationship between gut microbes and their hosts is characterized by intricate interdependencies (Linares et al., 2016). Additionally, gut microbes can generate various beneficial small-molecule metabolites that contribute to the host’s physiological activities (Krishnan et al., 2015). These metabolites participate in the host’s energy metabolism, modulate immune function and maintain the balance of the microbial community (Canfora et al., 2019; Garrett, 2020). Simultaneously, the host provides a stable and nourishing environment for the growth of gut microbes.

Although the term “core microbiome” is commonly used, there is still confusion and disagreement regarding its relevance in ecological and biological research. Frequently, the core microbiome pertains to the collection of microbial species or taxa that consistently inhabit a specific environment, sample, or group of organisms. While the overall composition of the microbiome may fluctuate among individual organisms due to factors like diet, environment, and genetics, the core microbiome constitutes a steady and enduring subset of microorganisms that play a role in the holistic functioning of the host organism (Turnbaugh et al., 2007; Tschöp et al., 2009; Kokou et al., 2019). The core microbiome plays a critical role in maintaining individual and gut health, significantly impacting the microbial community (Perlman et al., 2022). Microbial communities exhibit highly complex taxonomic diversity and there is a positive correlation between biodiversity and stability. In terms of maintaining microbial community stability, the impact of specific species outweighs the overall number of species in the community. Consequently, the core microbiome plays a crucial role in ensuring the functional stability of soil microbial communities (Xun et al., 2021; Jiao et al., 2022).

To date, limited studies have explored the core gut microbiome of mud crab. Understanding the core microbiome is essential for maintaining the stability and resilience of the host’s gut microbial community, as it is closely associated with beneficial functions for the host. Therefore, a comprehensive investigation of the gut microbiota of mud crab is necessary to identify its core gut microbes and fully comprehend the richness and significance of these microbes in the host’s health and ecosystem (Zhou et al., 2009; Sehnal et al., 2021; Sun and Xu, 2021; Zhang and Sun, 2022).

In this study, we utilized Illumina MiSeq’s 16S rRNA sequencing technology to examine the gut microbial composition, diversity and function of mud crab from multiple regions of Sanmen Bay. We observed variations in gut microbes based on sex. Moreover, we discovered the existence of a core mud crab gut microbiome with remarkably similar populations. Identifying these core microbiomes can assist in recognizing the assembly and drivers of microbial communities, thereby significantly enhancing our ability to manipulate the microbiome and address microbial perspectives to overcome challenges encountered in mud crab production activities.

## 2. Materials and methods

### 2.1. Sample collection

The experimental samples were collected from the seawater aquaculture ponds in Sanmen Bay (29°02′53″N, 121°42′38″E) and the sampling points were located in Huaqiao Town (HQ,28°92′26.45″N, 121°47′94.77″E), Jiantiao Town (JT, 29°04′46.52″N, 121°62′80.50″E), Pubagang Town (PBG, 28°96′52.32″N, 121°61′64.63″E), Shaliu Town (SL,29°15′07.39″N, 121°40′26.16″E) and Shepan Town (SP,29°13′66.16″N, 121°58′44.48″E; Supplementary Figure S1). Fifteen healthy male and female crabs (weighing 150–200 g) were selected from each sampling point. After the crabs were anesthetized with ice-cold water, their intestines and contents were immediately separated and the samples were frozen in liquid nitrogen for 16S rRNA gene sequencing. When extracting DNA and ten samples were collected from each sampling point, for a total of 50 samples.

### 2.2. DNA extraction, PCR amplification and Illumina MiSeq sequencing

The bacterial genomic DNA of the gut samples was extracted by DNeasy PowerLyzer PowerSoil Kit (QIAGEN) in accordance with the manufacturer’s protocol. The quality of extracted DNA was assessed with a NanoDrop 2000 instrument (Thermo Fisher Scientific, Wilmington, MA, United States) based on absorption ratios at 260/280 nm and 260/230 nm. The 16S rRNA genes were partially amplified with the bacterial universal V3-V4 primer 343F (5’-TACGGRAGGCAGCAG-3′) and 798R (5′- AGGGTATCTAATCCT-3′). A 30 μL polymerase chain reaction (PCR) system, including 11 μL ddH2O, 1.0 μL of each primer (10 mmol/L), 15 μL 2 × SYBR Prime-Script™ Master Mix (TaKaRa, Japan) and 2 μL of template DNA. The PCR was performed in a 30 μL volume with 15 μL of 2 × HiFi Hot Start Ready Mix, 1.0 μL of forward and reverse primers (10 μmol/L) and 50 ng of genomic DNA as template. Thermal cycling consisted of initial denaturation at 94°C for 5 min; followed by 26 cycles of denaturation at 94°C for 30 s, annealing at 56°C for 30 s, elongation at 72°C for 30 s and a final extension at 72°C for 7 min (Niu et al., 2023). Agencourt Ampure XP beads (Beckman, CA, United States) were used to purify PCR products. Following assessment of fragment size and quantification and then these amplicons were sequenced in equimolar and paired-end (2 × 300) using the Illumina MiSeq platform.

### 2.3. Processing of Illumina sequencing data

PE readings were preprocessed using Trimmomatic software (Bolger et al., 2014). After trimming, FLASH software was used to compile paired-end reads (Reyon et al., 2012). The Vsearch program was used to eliminate the primer sequences and cluster sequences into generated OTUs with a 97% similarity cut-off value (Rognes et al., 2016). The QIIME software suite was used to pick representative contigs for each OTU. The Silva database (version 138) was annotated and parsed using the RDP classifier (70% confidence threshold) (Wang, 2007).

### 2.4. Statistical analyses

Mothur software (version 1.32.1) was used to analyze Alpha diversity indexes, including Chao 1, observed species, Simpson, Shannon diversity and Good’s coverage estimate using an OTU specified at the 97% identity level (Schloss Patrick et al., 2009). Based on Binary-jaccard distance metrics, PCoA, UniFrac distance and ANOVA were performed to examine the overall differences in the bacterial community of the crab guts (Clarke, 1993). Additionally, heat map analysis was applied to analyze the differences in microorganisms in five groups. To explore the core gut microbial correlations among the five sample sites, the correlation matrix was plotted using R’s corrplot. Evaluate the stability of microbial communities by Average Variation Degree and correlate the stability with the relative abundance of core taxa. The remaining figures are generated by the R scripts.

### 2.5. Prediction of functional potentials

The PICRUSt2 (Douglas et al., 2020) was performed to predict the functional composition of known microbial genes based on 16S sequencing data annotated in the Greengenes database (Desantis et al., 2006), so as to count the functional differences between different groups. The KEGG pathways was retrieved according to KEGG database.^1^ Multiple comparisons were performed using the Kruskal–Wallis test, ggplots2 in R was used to construct the plots.

## 3. Results

### 3.1. OTUs and diversity analysis of intestinal microbiota in *Scylla paramamosain* from Sanmen Bay

In the present study, we examined the microbial community composition of the intestinal tract of *S. paramamosain* in five different sampling sites within Sanmen Bay. We employed the Illumina MiSeq PE300 platform for sequencing, which generated paired-end sequence data. A total of 3,818,498 raw 16S rRNA gene sequence tags were obtained from sequencing 50 samples. After filtering out low-quality and chimeric sequences, we retained 3,619,239 high-quality sequence tags for further analysis. Each sample consisted of 65,487–75,229 sequences, with sequence lengths ranging from 228 to 442 bp (Supplementary Figure S2). By applying a 97% sequence identity threshold, we identified a total of 5,773 operational taxonomic units (OTUs) through clustering the high-quality sequences (Supplementary Table S1). The rarefaction curve reached a saturation plateau (Supplementary Figure S3A), indicating that the sequencing depth was sufficient to obtain a stable and unbiased assessment of species abundance. The specaccum accumulation curve showed a gradual increase (Supplementary Figure S3C), suggesting that the sample size was adequate to reflect community richness. The Goods coverage index exceeded 99% in all samples (Supplementary Figure S3B), indicating that the sequencing depth covered all species in the samples and the sequences were valid. Each regional sample contained 599 to 1,262 OTUs. Among the five regional samples, there were 1,285 shared OTUs and each region had unique OTUs: 161 (SP), 259 (JT), 339 (PBG), 385 (HQ) and 803 (SL; Supplementary Figure S4).

**Figure 1:**
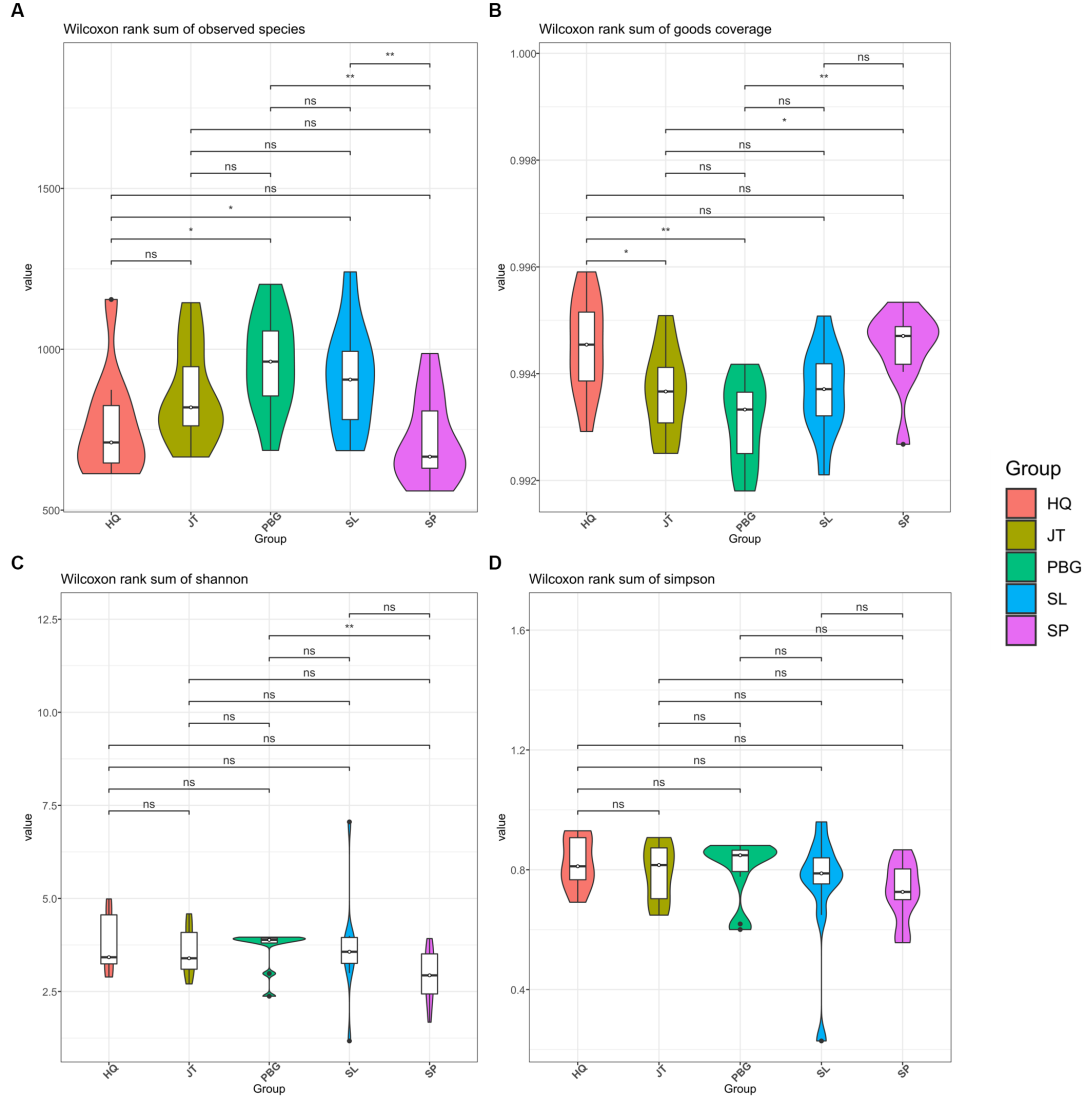
Alpha diversity of intestinal microbiota in *S. paramamosain*. **(A–D)** stand for Chao1, Observed species, Shannon and Simpson index, respectively.

We analyzed the alpha-diversity of the intestinal microbial communities in mud crabs across the five sampling sites using Chao1, Observed species, Shannon diversity and Simpson indices (Figure 1). The Chao1 index ranged from 1,024 to 1,824 and significant differences were observed between SP and SL, PBG and SL, as well as PBG and HQ (Figure 1A). Similarly, significant differences were found in Observed species between SP and JT, PBG and JT and PBG and HQ (*p* < 0.05), indicating variations in community richness among the different sampling sites. However, when assessing the Shannon diversity and Simpson indices, which evaluate the diversity of the intestinal microbial communities, only SP and PBG exhibited a significant difference in Shannon diversity (*p* < 0.05). Overall, there were no significant differences in the microbial community diversity of the intestinal tract among the five regions.

**Figure 2:**
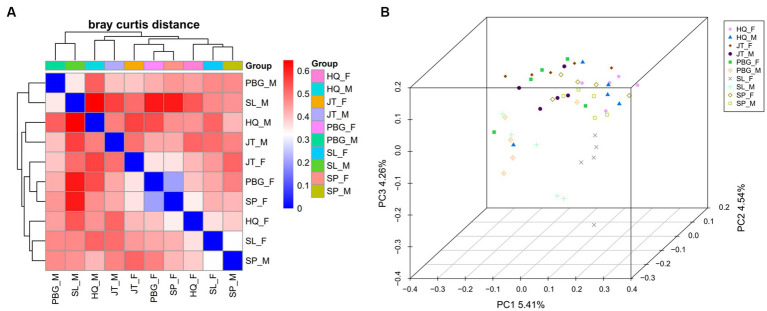
Beta diversity analysis of the intestinal microbial community. **(A)** Heatmap showing species abundance clustering of samples; **(B)** principal coordinates analysis of microbial communities of samples was based on the Binary jaccard.

Furthermore, heatmap clustering analysis and PCoA plot analysis (Figure 2) revealed both separation and overlapping of samples in each sampling point. Overall, dispersion is low and clustering is relatively concentrated. Notably, samples of the same sex from specific sampling sites tended to cluster together. This observation suggests that there may be gender-related differences in microbial community composition across sampling sites.

### 3.2. Composition of microbial communities in *Scylla paramamosain* from Sanmen Bay

All OTUs belong to 35 phyla, 94 classes, 245 orders, 406 families and 815 genera (Supplementary Table S2). At the phylum level, the gut microbiota community in the five sampling points of *S. paramamosain* was similar, but there were differences in relative abundance. Firmicutes (49.80%), Proteobacteria (22.49%), Bacteroidota (11.95%) and Campilobacterota (5.53%) were dominant in all samples (relative abundance >5% in all samples; Supplementary Figure S5A), representing the common dominant phyla of the mud crab gut microbiota in Sanmen Bay. Additionally, Spirochaetota (7.94%) had a relatively high relative abundance in JT. Campilobacterota was also dominant in PBG (7.29%) and SL (6.59%). SP had the highest number of dominant microbial phyla in the gut, including Fusobacteriota (6.63%), Campilobacterota (5.90%) and Spirochaetota (5.51%). Entotheonellaeota was only found in SL, although its relative abundance was very low. Among the top 15 phyla with relatively high abundance (Supplementary Figure S5A), Campilobacterota (M > F), Patescibacteria (M > F), Spirochaetota (M < F) and Acidobacteriota (M < F) showed significant differences in relative abundance between males and females (*p* < 0.05).

At the genus level, the top 15 genera with relatively high abundance in the samples were Photobacterium, Candidatus_Hepatoplasma, Carboxylicivirga, Defluviitaleaceae_UCG011, Vibrio, Sediminispirochaeta, Halarcobacter, ZOR0006, Hypnocyclicus, Psychrilyobacter, [Anaerorhabdus]_furcosa_group, Malaciobacter, Tepidibacter, Proteocatella and DesulfoVibrio (Supplementary Figure S5B). Except for DesulfoVibrio, all these genera were dominant in the gut microbiota of the mud crab in Sanmen Bay (relative abundance >1%), accounting for nearly half or more than half of the total sequences in the samples. Among them, Sediminispirochaeta, ZOR0006 and DesulfoVibrio showed significant differences in relative abundance among different sampling points (*p* < 0.05). The relative abundance of Sediminispirochaeta and ZOR0006 in HQ was lower than that in PBG, SP and JT and the relative abundance of DesulfoVibrio in HQ was significantly lower than in other sampling points. We also found significant differences in the relative abundance of Vibrio, Sediminispirochaeta, Hypnocyclicus, [Anaerorhabdus]_furcosa_group, Malaciobacter and Proteocatella between males and females in all samples (*p* < 0.05).

### 3.3. Core intestinal microbiota in *Scylla paramamosain* from Sanmen Bay

**Figure 3:**
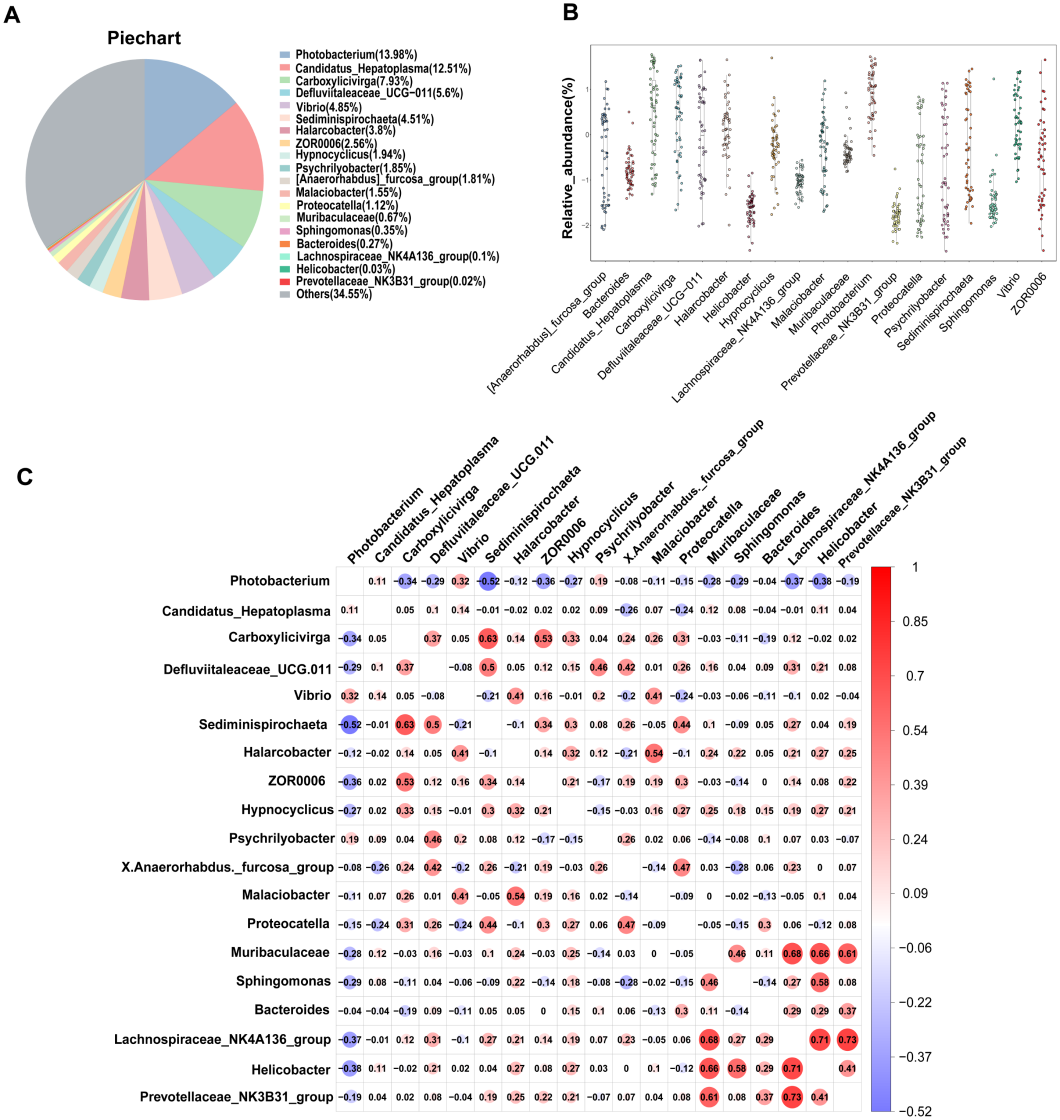
Core gut microbiota composed of 19 bacterial genera in *S. paramamosain* in Sanmen regions samples. **(A)** The proportion of each genus in all sequences combined. **(B)** The abundance and distribution of 19 core genera. **(C)** Correlation matrix showing the Spearman’s rank correlations among the collective core, which ranges from −1 to 1, corresponding to a strongly positive to a strongly negative correlation, respectively.

One of the primary objectives of this study was to examine whether the intestinal microbiota of *S. paramamosain* in Sanmen Bay shared a common core microbial community within the crab’s gut. Additionally, we aimed to shed light on the significance of this core intestinal microbiota. The concept of a core microbial community is typically defined by the presence of shared OTUs across all samples (Shade and Handelsman, 2012; Astudillo-García et al., 2017; Chen et al., 2021; Maji et al., 2022). In our study, we identified 34 core OTUs that were present in all samples, thus considered as candidate core taxa (Supplementary Figure S6). These core OTUs were further classified at the genus level, resulting in 19 candidate core genera (Table 1). These 19 genera accounted for 65.45% of the total sequences (Figure 3A) and were distributed across six phyla, with Firmicutes and Proteobacteria representing 42.91% of the genera, while the remaining genera belonged to Bacteroidota, Campilobacterot, Fusobacteriota and Spirochaetota. Although the 19 core genera were detected in all samples, their relative abundances varied significantly among the samples (Figure 3B; Table 1). Additionally, we examined the co-occurrence patterns among these genera using Spearman’s rank correlation (Figure 3C). The results demonstrated that *Photobacterium* exhibited predominantly negative correlations with other genera (ρ = −0.52–0.32), particularly *Sediminispirochaeta* (ρ = −0.53). Conversely, *Prevotellaceae_NK3B31_group* exhibited predominantly positive correlations with other genera (ρ = −0.19–0.73), especially *Lachnospiraceae_NK4A136_group* (ρ = 0.73). Moreover, *Helicobacter* displayed strong positive correlations with *Lachnospiraceae_NK4A136_group*, Sphingomonas and Muribaculaceae (ρ = 0.71–0.58). Overall, except for certain core genera, most of the core genera exhibited varying degrees of positive correlations, suggesting a predominantly symbiotic interaction among the core taxa.

**Table 1:** The core genera identified in samples.

Phylum	Genus	Relative abundance (%)	Range (%)
Bacteroidota	*Carboxylicivirga*	7.93	0.02–33.03
Bacteroidota	Muribaculaceae	0.67	0.15–8.38
Bacteroidota	Bacteroides	0.27	0.04–3.16
Bacteroidota	Prevotellaceae_NK3B31_group	0.02	0.00–0.17
Campilobacterota	*Halarcobacter*	3.80	0.01–44.80
Campilobacterota	*Malaciobacter*	1.55	0.02–15.36
Campilobacterot	Helicobacter	0.03	0.00–0.14
Firmicutes	*Candidatus_Hepatoplasma*	12.51	0.05–58.27
Firmicutes	*Defluviitaleaceae_UCG-011*	5.60	0.01–44.24
Firmicutes	*ZOR0006*	2.56	0.00–44.76
Firmicutes	*[Anaerorhabdus]_furcosa_group*	1.81	0.01–14.82
Firmicutes	*Proteocatella*	1.12	0.01–6.80
Firmicutes	Lachnospiraceae_NK4A136_group	0.10	0.02–0.27
Fusobacteriota	*Hypnocyclicus*	1.94	0.02–49.45
Fusobacteriota	*Psychrilyobacter*	1.85	0.00–13.65
Proteobacteria	*Photobacterium*	13.98	0.35–52.43
Proteobacteria	*Vibrio*	4.85	0.09–24.83
Proteobacteria	Sphingomonas	0.35	0.01–16.97
Spirochaetota	*Sediminispirochaeta*	4.51	0.01–28.16

Furthermore, when analyzing the samples from male and female crabs, differences in the core OTUs between the sexes were observed. There were 3,516 shared OTUs between male and female samples, with 1,292 and 965 OTUs being unique to females and males, respectively (Supplementary Figure S7A). The core OTUs consisted of 58 for males and 45 for females (Supplementary Figure S8), which encompassed 30 and 22 genera at the genus level, respectively. Among these, 16 core genera were shared between both sexes. Males exhibited 12 unique core genera, belonging to the phyla Bacteroidota, Firmicutes and Proteobacteria including *ParaBacteroides*, *Rikenellaceae_RC9_gut_group*, *Muribaculum*, *Colidextribacter*, *Blautia* and other genera. In contrast, females had only 2 unique core genera, both belonging to the phylum Bacteroidota, namely *Sunxiuqinia* and *Alloprevotella* (Supplementary Figure S7B). These findings suggest that there are distinct differences in the core gut microbial communities at the genus level between male and female crabs.

### 3.4. Association between core microbiota and intestinal microbiome stability

**Figure 4:**
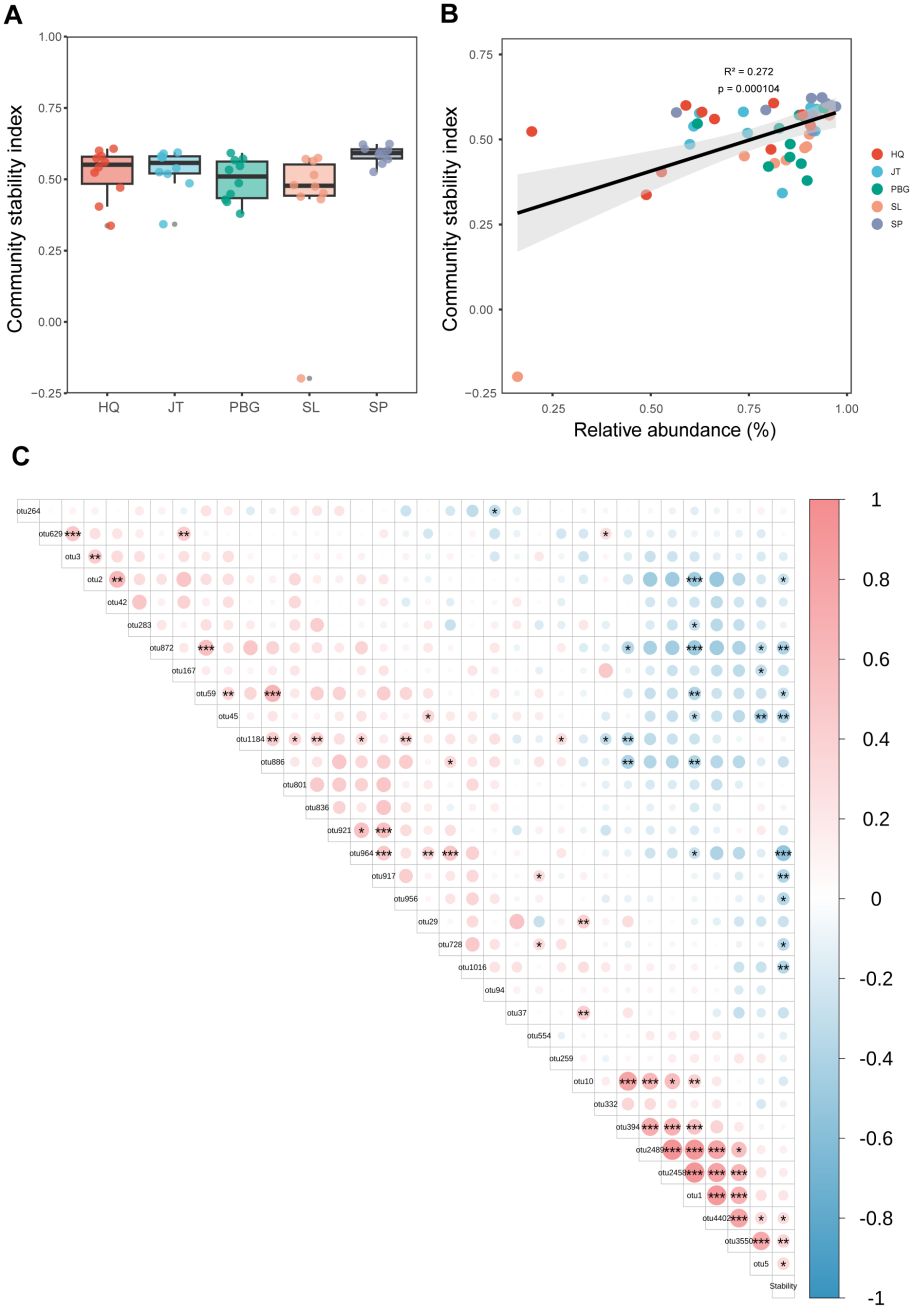
Association between core microbiota and gut microbiome stability. **(A)** Stability of the *S. paramamosain* gut microbiome. **(B)** Linear relationship between the relative abundance of core gut microbiota and community stability index. **(C)** Relationship between the relative abundance of core OTU and the community stability index stability. *, ** and *** represent *p* < 0.05, *p* < 0.01 and *p* < 0.001, respectively.

To assess the stability of the intestinal bacterial community, we utilized the Average Variation Degree (AVD) index. This method is commonly used to estimate the stability of microbial community structure (Xun et al., 2021; Jiao et al., 2022). Our findings revealed differences in the stability of the intestinal microbial community structure among the different sampling points, with significant variations observed between SP and PBG, as well as SL (*p* < 0.05) (Figure 4A). Furthermore, we investigated the correlation between core intestinal microbes and microbial community stability. Interestingly, we found a positive correlation between the relative abundance of core OTUs and microbial community stability (*p* < 0.01; Figure 4B). This indicates that the core microbial group may play a crucial role in maintaining the stability of the intestinal microbial community. Specifically, OTU5, OTU3550 and OTU4402 (*p* < 0.05; Figure 4C) were identified as significant contributors to the stability of the intestinal microbial community.

### 3.5. Predicted function of intestinal microbiota community

**Figure 5:**
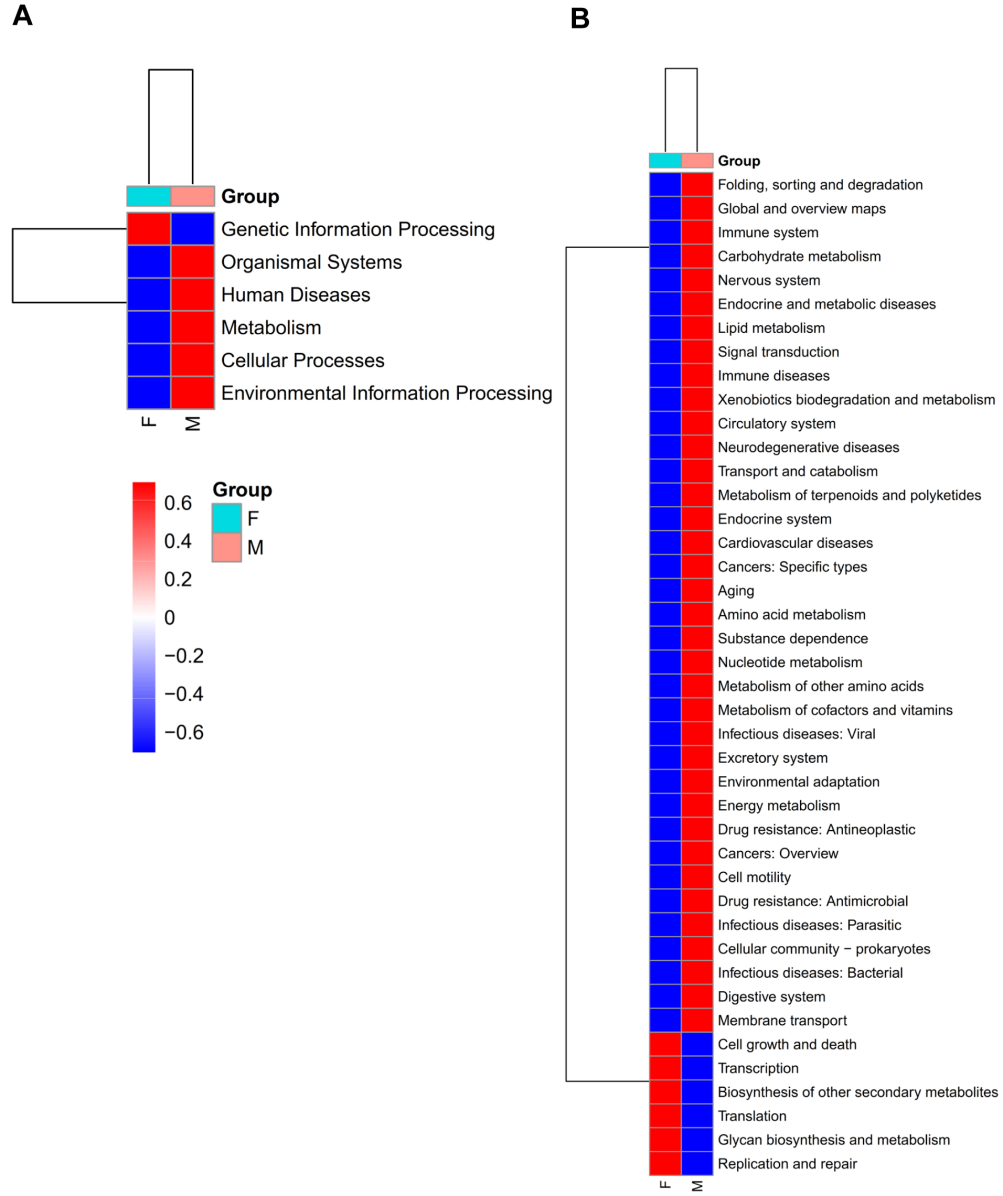
At L1 **(A)** and L2 **(B)** level, heat map of KEGG function prediction of core gut microbial of the females and males groups. Red indicates a higher relative abundance of species and blue indicates a lower relative abundance.

To assess the functional potential of the mud crab’s intestinal microbiota, we conducted functional predictions of the core and non-core microbial communities using PICRUSt2 (Supplementary Figure S9; Figure 5). The analysis showed that the core gut microbiota had a total of 42 predicted functional pathways at the L2 level, whereas the non-core microbiota predicted a total of 46 functional pathways. These pathways primarily encompassed Global and overview maps, Carbohydrate metabolism, Amino acid metabolism, Energy metabolism and Membrane Transport. The non-core microbial community included all the functional pathways present in the core microbial community and additionally exhibited pathways such as Cellular community – eukaryotes, Development and Sensory system. Furthermore, we observed differences in the enrichment levels of shared functional pathways, indicating distinct distributions and enrichment patterns of functional pathways between the two communities. This highlights the diversity and complexity of the mud crab’s intestinal microbial community.

Moreover, significant differences were identified in the enrichment levels of functional pathways between male and female crabs, both in the core and non-core microbial communities. The core microbial community of males exhibited higher enrichment levels in most functional pathways compared to females, while the non-core microbial community of females demonstrated higher enrichment levels in most functional pathways compared to males. These findings strongly suggest that gender plays a significant role in shaping the structure and functional pathways of the mud crab’s intestinal microbial community.

## 4. Discussion

The present study, we observed no significant differences in the diversity of the intestinal microbial community among the five sampling sites in Sanmen Bay’s mud crab. However, variations in community richness were evident across different sampling sites (Figure 1). At the phylum level, the dominant bacterial phyla in the gut microbial community of mud crabs were Firmicutes, Proteobacteria, Bacteroidota and Campilobacterota (Supplementary Figure S5). These phyla are commonly found in the gut microbiota of aquatic invertebrates, including mud crabs, which share similar gut microbial community compositions (Wei et al., 2019; Chen et al., 2021; Ma et al., 2021). Campilobacterota is widely present in the oral and intestinal microbiota of both vertebrates and some invertebrates, highlighting its importance in various animal gut microbial communities (Ma et al., 2020; Farag et al., 2022). Firmicutes and Bacteroidota play crucial roles in the growth, digestion, metabolism and immune function of aquatic animals (Foysal et al., 2021; Yang et al., 2023). At the genus level, we identified 19 core genera (Table 1) primarily engaged in symbiotic interactions (Figure 3C). These core genera accounted for 65.45% of the total sequences and were distributed across six phyla, including the dominant phyla mentioned earlier, as well as Fusobacteriota and Spirochaetota. Comparing the core genera identified in this study with the research by Wei et al. (2019), we found that *Candidatus_Hepatoplasma*, *Vibrio*, *Bacteroides*, *Photobacterium* and *Carboxylicivirga* were also identified as core genera in the gut microbiota of mud crab from different coastal regions in southern China. These core gut microbiota members may have established stable mutualistic symbiotic relationships with the mud crab (Oku et al., 2008; Leclercq et al., 2014; de Souza Valente and Wan, 2021). Furthermore, the core genera in mud crabs from Sanmen Bay exhibited quantitative and compositional differences compared to those in other coastal regions in southern China, suggesting potential geographical variations in the core gut microbiota of mud crab. This finding implies that the gut microbial community structure of mud crab may be influenced by geographical factors.

Significant differences exist in the average relative abundance of core genera within the intestinal microbiota of mud crabs in Sanmen Bay. Among these genera, *Photobacterium* exhibits the highest average relative abundance (Figures 3A,B). *Photobacterium* is a marine bacterium belonging to the *Vibrio*naceae family, commonly found in the intestines of fish and other marine organisms (Moi et al., 2017). It possesses the capability to produce various beneficial substances, including polyunsaturated fatty acids, cold-adapted lipase, asparaginase, esterases and antimicrobial compounds (Le Doujet et al., 2019). Previous studies have demonstrated that certain strains of *Photobacterium* exhibit probiotic activity in animal models. They promote the growth of beneficial bacterial communities, inhibit the growth of pathogenic bacteria, enhance the host’s immune response and maintain intestinal health (Oku et al., 2008; Urbanczyk et al., 2011). Consequently, it is possible that *Photobacterium* may play a similar role in promoting the growth of beneficial bacterial communities in the mud crab intestine, inhibiting the growth of pathogens, enhancing the host’s immune response and maintaining intestinal microbiota diversity. However, it is important to note that these functions have not been specifically studied or validated in the context of mud crabs and therefore require further investigation.

The core microbial taxa have a significant influence on the formation and stability of the intestinal microbial community (Lemanceau et al., 2017; Jiao et al., 2022). In this study, we observed significant differences in the stability of the intestinal microbial community among different sampling sites in Sanmen Bay (SP and SL, PBG) (Figure 4A). This suggests that the stability of the gut microbiota is influenced by geographical location, possibly due to variations in environmental conditions among different regions (Supplementary Table S3). Moreover, we found a significant positive correlation between core microbial taxa and the stability of the gut microbiota (Figure 4), indicating the crucial role of these core taxa in maintaining gut microbiota stability. Further analysis identified OTU5, OTU3550 and OTU4402 as the core taxa most significantly positively correlated with gut microbiota stability (*p* < 0.05; Figure 4C). OTU5 belongs to Firmicutes, although its genus level has not been identified; OTU3550 and OTU4402 belong to Proteobacteria and *Photobacterium*, respectively, at the phylum and genus levels. Bacteria belonging to the Firmicutes are known to produce a wide range of digestive enzymes, facilitating host digestion and nutrient absorption (Hudson and Egan, 2022). Proteobacteria dominate the gut microbiota of aquatic crustaceans (Ding et al., 2017; Apine et al., 2021) and have the ability to synthesize essential micronutrients like vitamin K2. They also regulate gut immunity by enhancing immune cell production and producing beneficial short-chain fatty acids, which contribute to energy metabolism and intestinal health (Saqib et al., 2023). Bacteria within the *Photobacterium* genus possess various potential functionalities, including the production of antimicrobial compounds and the promotion of beneficial bacterial growth, exhibiting probiotic activities (Cheung et al., 2015; Zhang et al., 2020). Therefore, the core intestinal microbiota may have the potential to regulate the intestinal microbial community in mud crab, enhancing the immune response and adaptability of the crabs. These findings reveal the significant role of core microorganisms in maintaining the stability of the intestinal microbial community, providing valuable insights into understanding the ecological functions of the intestinal microbiota and improving gut health.

Furthermore, we observed differences in the composition of the gut microbiota and the relative abundance of specific species between different sexes of the mud crab, as reflected in the core genera (Supplementary Figures S7, S8). While the influence of sex on the gut microbiota of aquatic animals is relatively small compared to environmental and genetic factors, it should not be ignored. Studies have shown that the gut microbiota structure and composition can vary between male and female fish, possibly due to metabolic differences between the sexes (Li et al., 2016). Similarly, the diversity of the gut microbiota in male wild Chilean octopus (*Octopus mimus* Gould, 1852) is higher than that in females, which may be related to their different feeding habits (Iehata et al., 2015). Gender has also been found to play a dominant role in shaping the functional aspects of the fish gut microbiota, as observed in *Betta splendens* (Gruneck et al., 2022). In our study, we observed that similar sex differences existed in the gut microbiota of mud crab, which are similar to previous findings in *Eriocheir sinensis* (Ma et al., 2021). The differences in gut microbiota between male and female crabs may be related to developmental disparities, which can impact the structure of the gut microbiota and subsequently influence the development of mud crabs. This intriguing observation warrants further investigation to elucidate the underlying mechanisms and potential implications for mud crab aquaculture. Overall, while sex may have a relatively minor influence on the gut microbiota compared to environmental and genetic factors, our study highlights the importance of considering sex as a factor that can contribute to variations in the gut microbiota composition in mud crabs. Further research is needed to fully understand the specific mechanisms driving these sex-related differences and their implications for the health and development of mud crabs in aquaculture settings.

The functional prediction of the core and non-core microbiota in the gut of mud crab in Sanmen Bay was conducted using PICRUSt2, revealing valuable insights into the potential functions and roles of the gut microbiota in this species. The functional pathways primarily involved Global and overview maps, Carbohydrate metabolism, Amino acid metabolism, Energy metabolism and Membrane Transport. These findings suggest that the gut microbiota plays a crucial role in nutrient utilization, metabolism, growth rate enhancement and environmental adaptability of the mud crabs. Furthermore, significant differences in the enrichment levels of functional pathways were observed between males and females in both the core and non-core microbiota. This indicates that gender influences the functional profiles of the microbial community, aligning with previous studies on gut microbiota and gender in other organisms (Mueller et al., 2006; Gomez et al., 2012; Markle et al., 2013; Abeles et al., 2014). However, limited research has explored the effects of gender on gut microbiota in crustaceans, highlighting the need for further investigations in this area. The composition and functional analysis of the gut microbiota in mud crab provide valuable insights into the impact of the microbiota on the health and growth of this species. In summary, this study successfully identified the core gut microbiota shared among mud crab in Sanmen Bay. These core microbial communities have been found to play a crucial role in the growth, immune function, and environmental adaptation of the crabs. Importantly, these findings hold promise for regulating the gut microbiota and enhancing the adaptability of mud crab in aquaculture settings. The discovery of these core microbial communities has significant biological implications, underscoring the importance of understanding their role in the gut of mud crab. By further elucidating the interactions between the host and its core gut microbiota, we can unlock new avenues for improving the health and performance of these commercially important crustaceans.

## 5. Conclusion

We conducted a comprehensive analysis of the structure, diversity, functionality, and stability of the gut microbiota in *S. paramamosain* from Sanmen Bay, China. We identified the core microbiome in this context. The clustering analysis yielded a total of 5,773 Operational Taxonomic Units (OTUs), with 34 of them classified as core OTUs. Within these 34 core OTUs, we identified 19 core genera. These 19 core genera account for 65.45% of the total sequences and span across 6 phyla. Among the core genera, *Photobacterium* had the highest average relative abundance and possessed probiotic activity. This suggests that it is highly adaptable to its host and has the potential to enhance immunity and maintain gut health in *S. paramamosain*. Moreover, our investigation revealed gender-specific differences in the composition and relative abundance of specific taxa, as well as the functionality of the gut microbiota in *S. paramamosain*. These differences may be attributed to variations in developmental processes and metabolic activities that occur between males and females. Furthermore, it is noteworthy that the core microbial group plays a vital role in maintaining the stability of the gut microbial community.

## Data availability statement

The original contributions presented in the study are included in the article/Supplementary material, further inquiries can be directed to the corresponding author.

## Ethics statement

The requirement of ethical approval was waived by the committee on the Ethics of Animal Experiments of Ningbo University (no. SYXK20190005) for the studies involving animals, because the animal subjects used in the present study are crabs, which are invertebrates and are exempt from this requirement. The studies were conducted in accordance with the local legislation and institutional requirements.

## Author contributions

HW and XJ conceived and designed the study and wrote the manuscript with support from all authors. XJ, MN, KQ, YH, YL, CC, CW, and CM performed all other experiments and analyzed data. All authors contributed to the article and approved the submitted version.

## Funding

This research was supported by the National Natural Science Foundation of China (42276106), the Major Science and Technology Innovation Tackling Project of Wenzhou (ZF2022008), the Key Scientific and Technological Grant of Zhejiang for Breeding New Agricultural Varieties (2021C02069-6), the earmarked fund for CARS (CARS-48), ZheJiang Agricultural Science and Technology Cooperation Project (2021SNLF029), and K. C. Wong Magna Fund in Ningbo University.

## Conflict of interest

The authors declare that the research was conducted in the absence of any commercial or financial relationships that could be construed as a potential conflict of interest.

## Publisher’s note

All claims expressed in this article are solely those of the authors and do not necessarily represent those of their affiliated organizations, or those of the publisher, the editors and the reviewers. Any product that may be evaluated in this article, or claim that may be made by its manufacturer, is not guaranteed or endorsed by the publisher.

## Footnotes

^1^http://www.kegg.jp/
