# Supplementary material for: The shared microbiome in mud crab (Scylla paramamosain) of Sanmen Bay, China: core gut microbiome
Source: Front Microbiol. 2023 Sep 1;14:1243334. doi: 10.3389/fmicb.2023.1243334 (PMC10505715; doi:10.3389/fmicb.2023.1243334)
Supplement: Supplementary file 1 [file Data_Sheet_1.docx]

**Table S1. Overview of sequencing data and alpha-diversity of samples from five areas in Sanmen Bay**

| Sample ID | | OTUs | | Shannon | | Simpson | | Goods coverage | | Chao1 | |
| --- | --- | --- | --- | --- | --- | --- | --- | --- | --- | --- | --- |
| HQ-F | 760.6 ± 89 | | 3.71 ± 0.92 | | 0.83 ± 0.101 | | 0.994 | | 1318.2 ± 177.7 | |  |
| HQ-M | 832.8 ± 228 | | 3.87 ± 0.77 | | 0.82 ± 0.075 | | 0.995 | | 1265.5 ± 287.4 | |  |
| JT-F | 913.6 ± 203 | | 3.65 ± 0.58 | | 0.81 ± 0.082 | | 0.994 | | 1476.4 ± 238.5 | |  |
| JT-M | 888.6 ± 113 | | 3.49 ± 0.73 | | 0.77 ± 0.114 | | 0.994 | | 1505.7 ± 137.6 | |  |
| PBG-F | 975.2 ± 203 | | 3.41 ± 0.70 | | 0.76 ± 0.135 | | 0.993 | | 1595.7 ± 174.9 | |  |
| PBG-M | 1034.4 ± 109 | | 3.90 ± 0.06 | | 0.85 ± 0.041 | | 0.993 | | 1632.5 ± 122.1 | |  |
| SL-F | 948.4 ± 180 | | 3.82 ± 2.11 | | 0.71 ± 0.279 | | 0.994 | | 1531.1 ± 127.1 | |  |
| SL-M | 969.2 ± 158 | | 3.54 ± 0.41 | | 0.79 ± 0.090 | | 0.994 | | 1521.7 ± 196.9 | |  |
| SP-F | 803.4 ± 169 | | 3.23 ± 0.63 | | 0.76 ± 0.113 | | 0.994 | | 1306.1 ± 233.9 | |  |
| SP-M | 714.4 ± 100 | | 2.63 ± 0.68 | | 0.70 ± 0.085 | | 0.995 | | 1244.4 ± 135.4 | |  |

**Table S2. Number of bacterial taxonomic units**

| Sample ID | Number of taxonomic units | | | | |
| --- | --- | --- | --- | --- | --- |
|  | Phylum | Class | Order | Family | Genus |
| HQ-F | 19.6±0.92 | 35±1.70 | 85.6±1.91 | 128.2±4.83 | 204.2±8.28 |
| HQ-M | 19.8±1.15 | 37.8±3.59 | 96.2±7.32 | 145.8±11.15 | 229.4±17.82 |
| JT-F | 20.4±1.98 | 35.6±4.86 | 94.0±9.69 | 144.0±13.62 | 218.4±22.01 |
| JT-M | 20.8±1.15 | 36.8±2.41 | 93.8±3.46 | 143.0±4.33 | 221.4±6.42 |
| PBG-F | 22.2±1.28 | 38.2±3.07 | 100.8±8.02 | 152.2±12.78 | 237.8±19.15 |
| PBG-M | 21.2±1.31 | 37.0±3.64 | 100.2±7.35 | 155.4±10.66 | 261.2±19.65 |
| SL-F | 20.6±2.03 | 39.2±5.43 | 96.0±8.55 | 141.6±13.09 | 210±15.67 |
| SL-M | 20.4±1.40 | 36.6±2.61 | 93.0±4.61 | 143.4±6.85 | 232.8±13.30 |
| SP-F | 18.2±0.37 | 32.4±1.88 | 85.4±3.14 | 130.4±6.72 | 197.4±14.35 |
| SP-M | 17.8±0.48 | 32.6±0.50 | 85.6±2.61 | 130.8±5.67 | 198.8±8.87 |
| Total | 35 | 94 | 245 | 406 | 815 |
| Total-F | 35 | 93 | 227 | 371 | 703 |
| Total-M | 34 | 81 | 216 | 359 | 717 |

**Table S3 Water quality parameters information in this study**

| Group | Salinity (‰） | T (℃) | pH | DO  (mg·L^-1^) | N  (mg·L^-1^) | AN  (mg·L^-1^) |
| --- | --- | --- | --- | --- | --- | --- |
| HQ | 17.82 ± 1.61 | 27.72 ± 2.63 | 8.21 ± 0.15 | 8.25 ± 0.84 | 0.52 ± 0.11 | 0.11 ± 0.05 |
| JT | 17.01 ± 2.02 | 27.21 ±3.13 | 8.23 ± 0.13 | 7.26 ± 0.87 | 0.76 ± 0.19 | 0.14 ± 0.06 |
| PBG | 23.2 ± 2.76 | 27.38 ± 3.87 | 8.45 ± 0.25 | 5.17 ± 0.87 | 0.79 ± 0.09 | 0.07 ± 0.05 |
| SL | 18.41± 1.22 | 27.35 ± 3.57 | 8.57 ± 0.14 | 7.99 ± 0.89 | 0.10 ± 0.02 | 0.10 ± 0.06 |
| SP | 24.6 ± 0.42 | 27.78± 2.54 | 8.41 ± 0.15 | 8.25 ± 0.84 | 0.52 ± 0.11 | 0.04 ± 0.02 |

**(T: temperature, DO: dissolved oxygen, N: nitrite, AN: ammonia nitrogen.)**

**Fig. S1 Samples collection in five regions of the San men country; HQ (Huaqiao Town), JT (Jiantiao Town), PBG (Pubagang Town), SL (Shaliu Town), and SP (Shepan Town)**

**
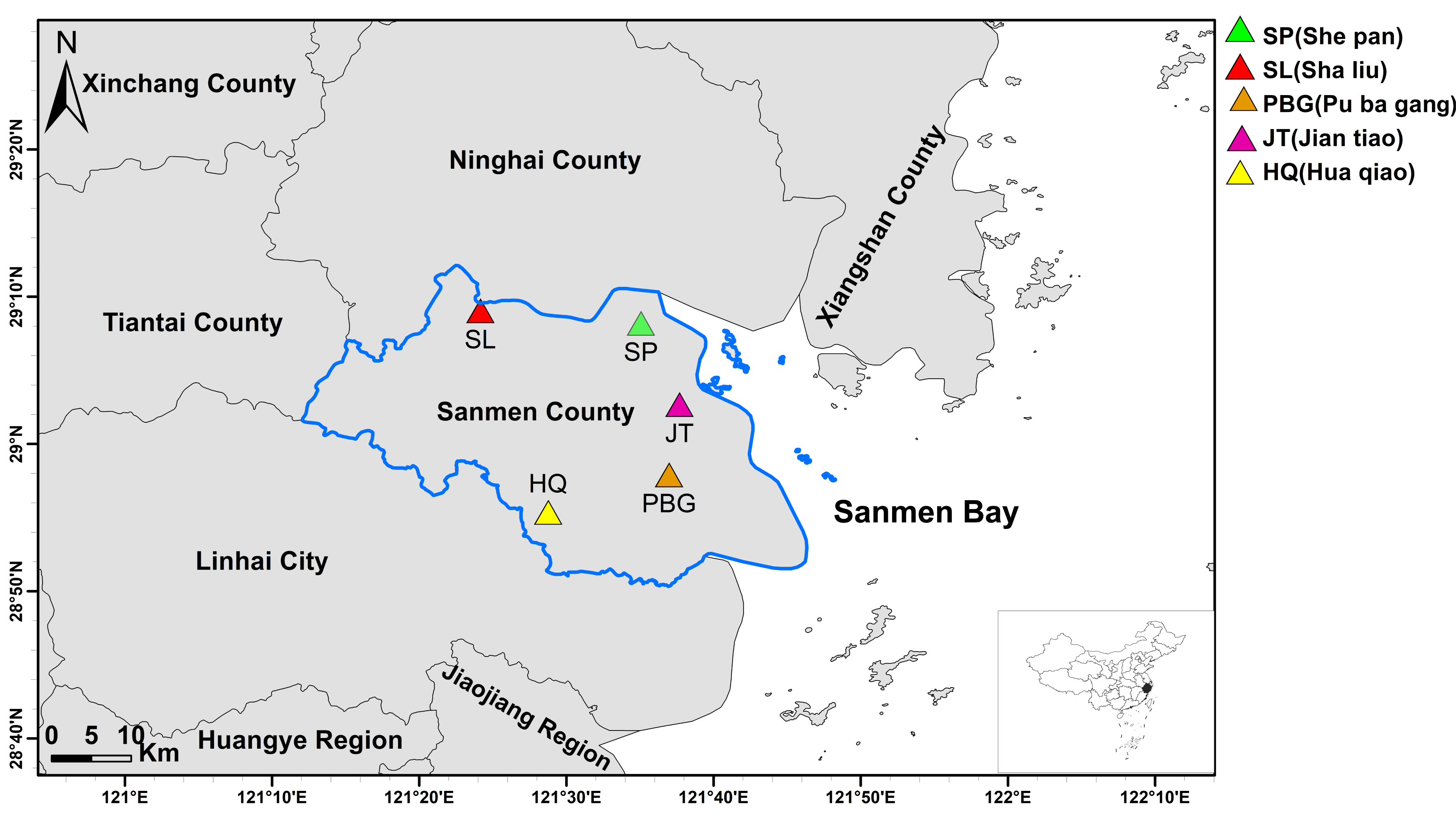
**


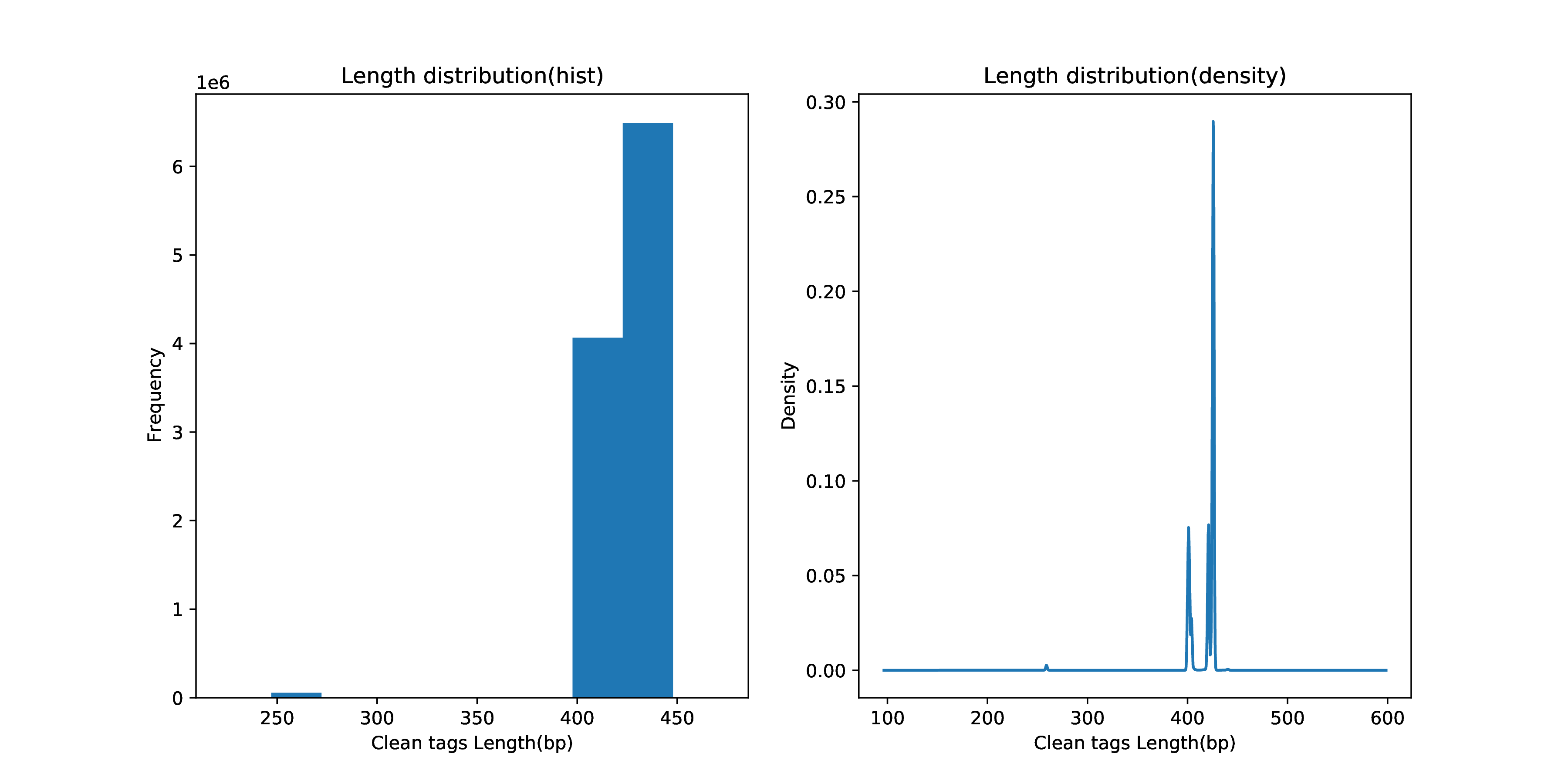
**Fig. S2 Distribution of raw sequences length**

**Fig. S3 Impact of sequening deth and sampling on bacterial phylotypes detection**


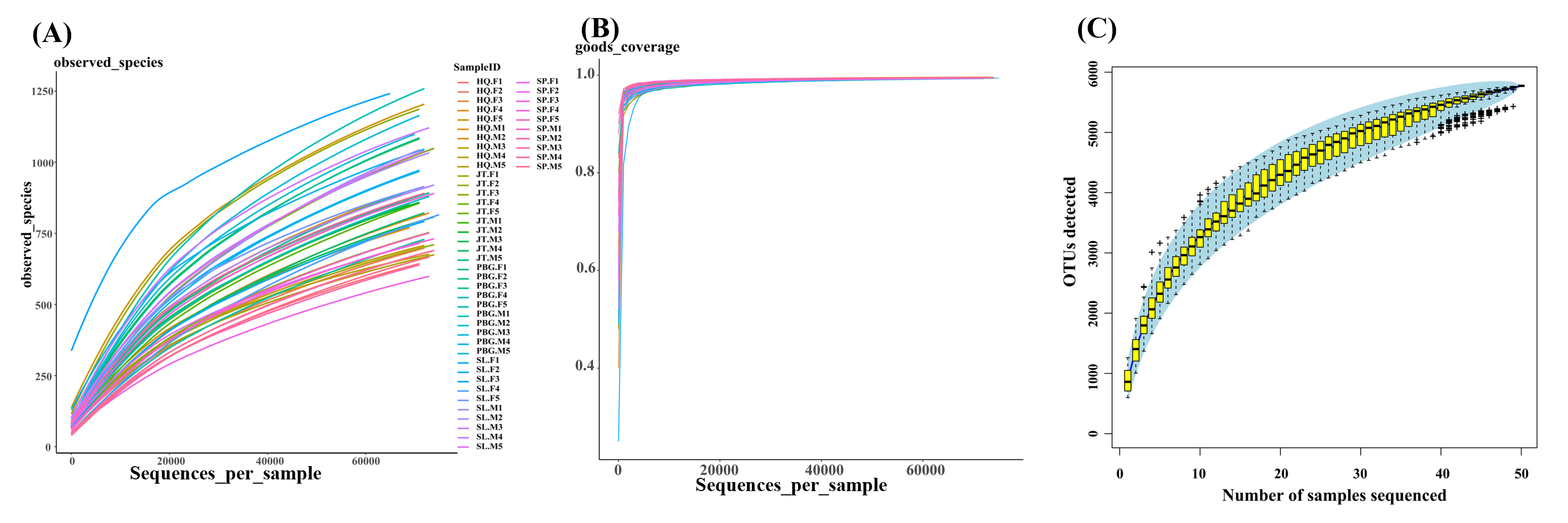


**Fig. S4** ***Scylla paramamosain* intestinal microbial Venn Diagram of OTUs in Sanmen area**


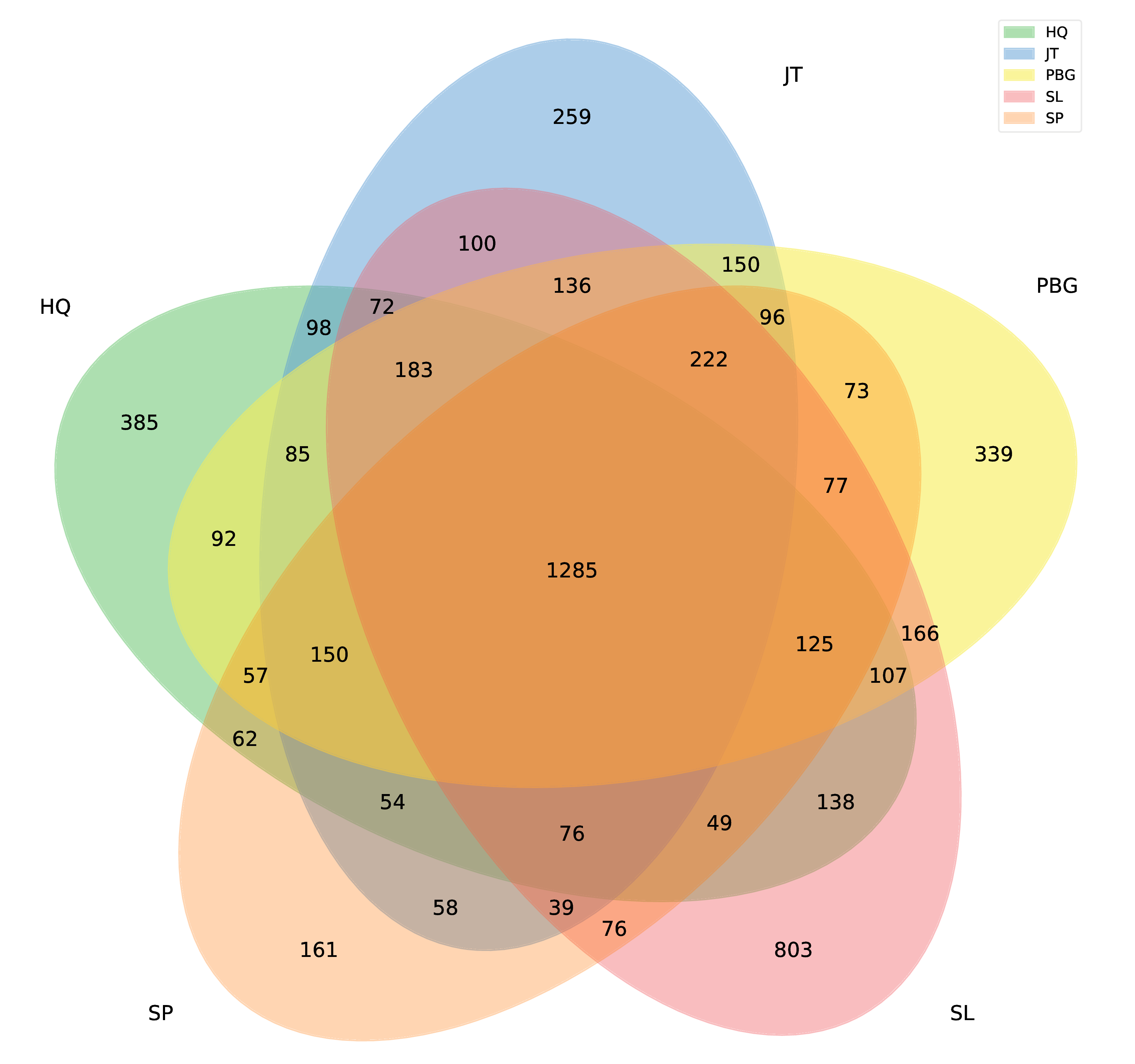


**Fig. S5 The bacterial community composition at the phyla (A), and genus (B) level in all samples. Figure shows the top 15 categories of relative abundance**

**
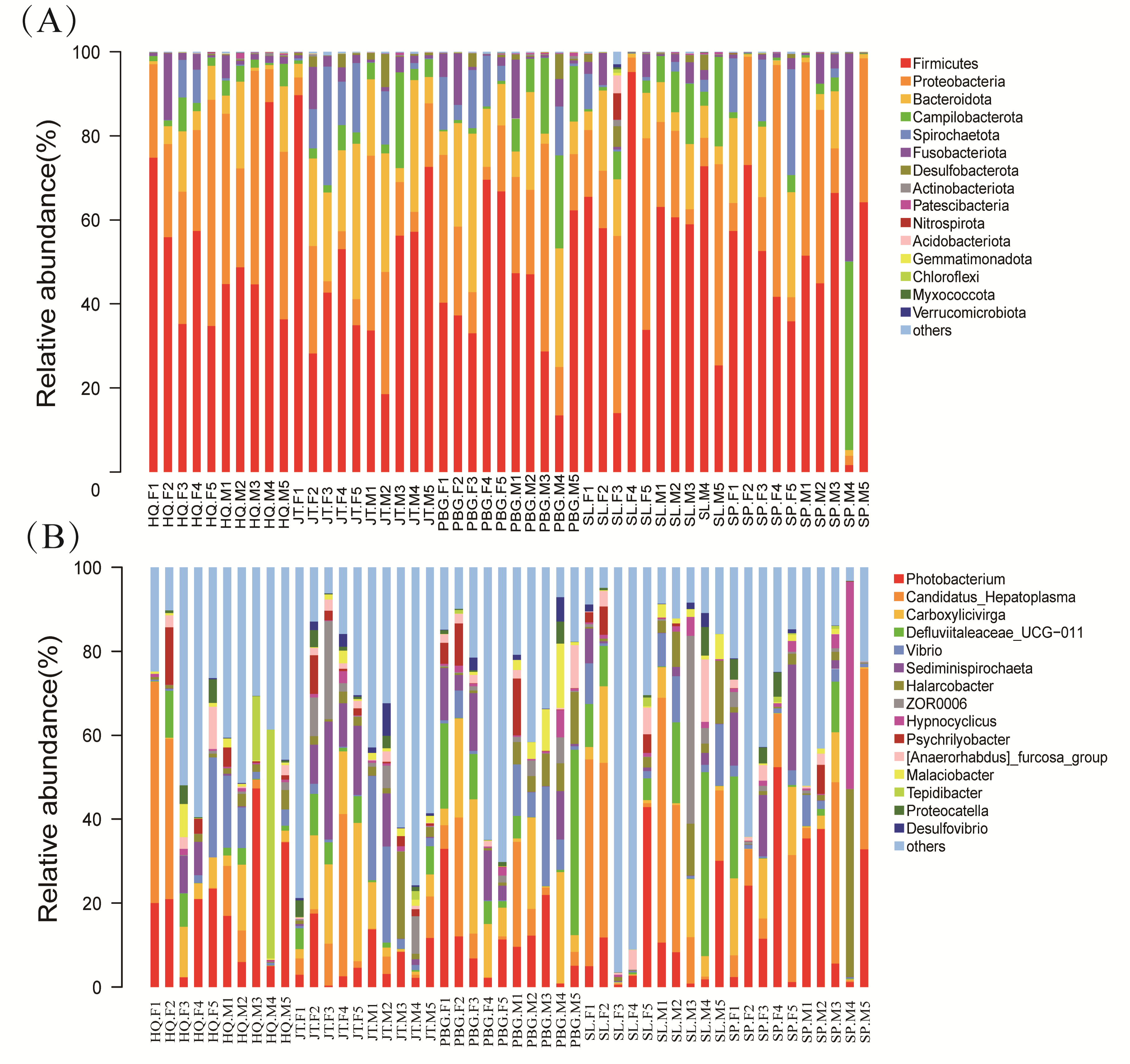
**

**Fig. S6 The core OTUs of samples**

**

–**

**Fig. S7 OTUs identified in male and female samples(A)；core genus identified in male and female samples(B)**

**(A) (B)**


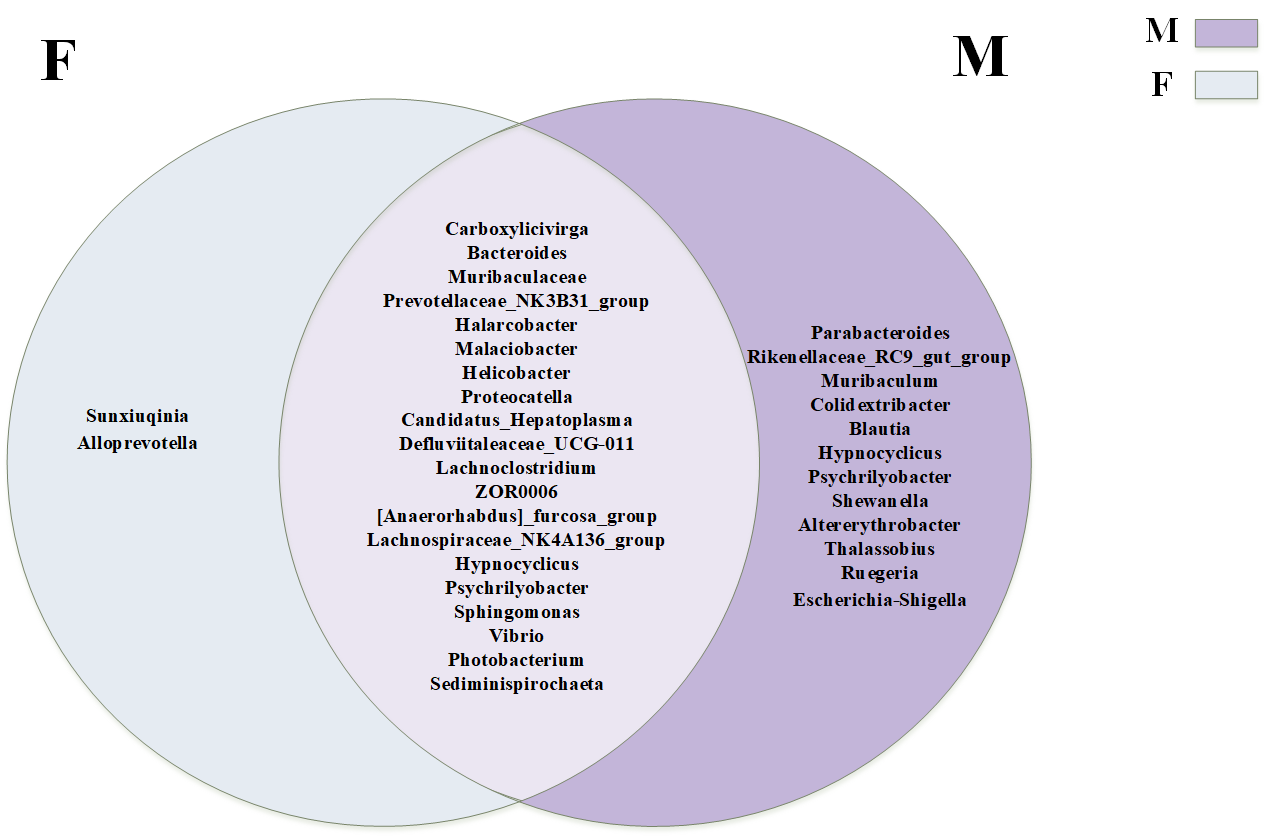

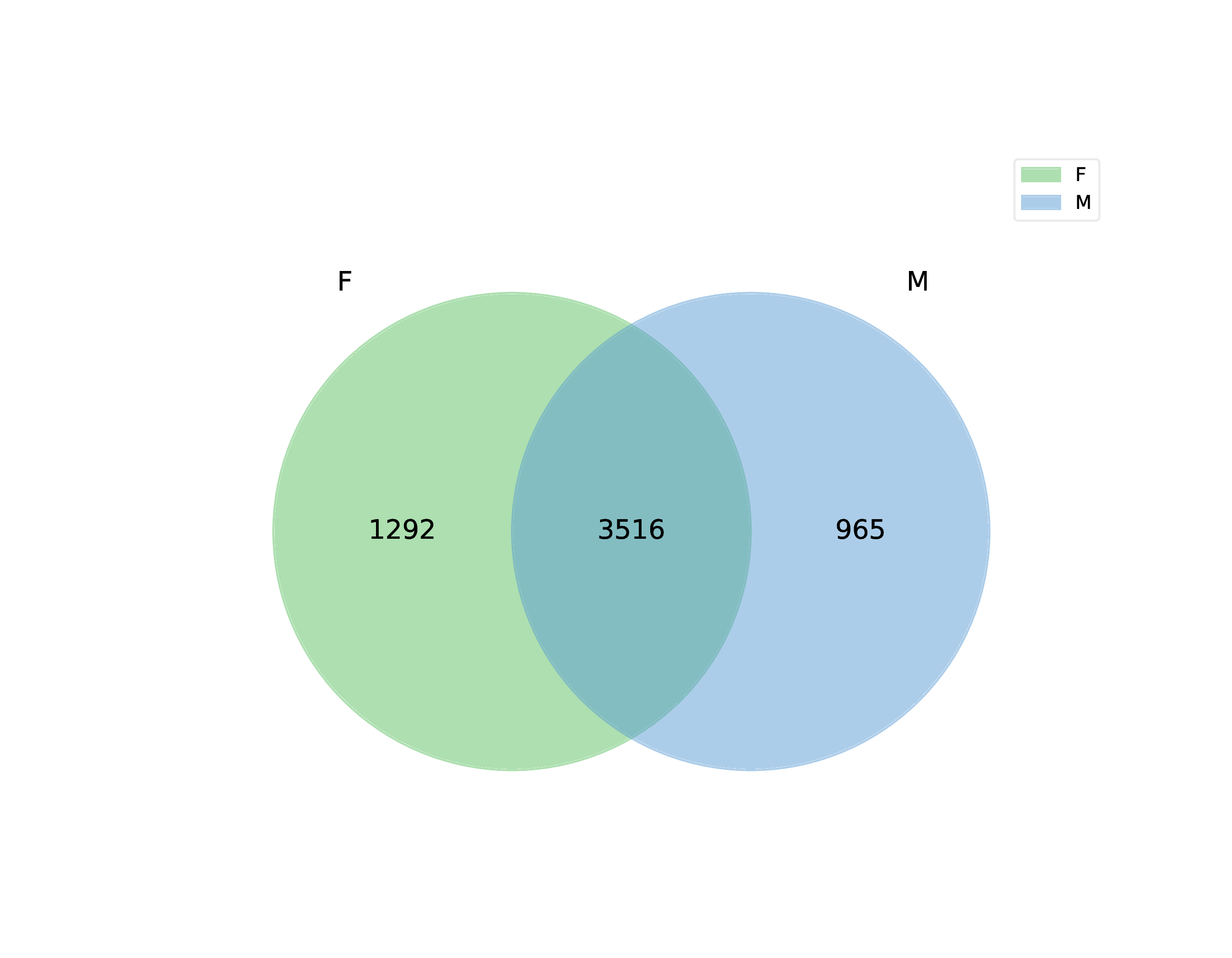


**

Fig. S8 Core OTUs that are different for females and males**

**Fig. S9 At L1 (A) and L2 (B) level, Heat map of KEGG function prediction of non-core gut microbial of the Females and Males groups. Red indicates a higher relative abundance of species and blue indicates a lower relative abundance**

**
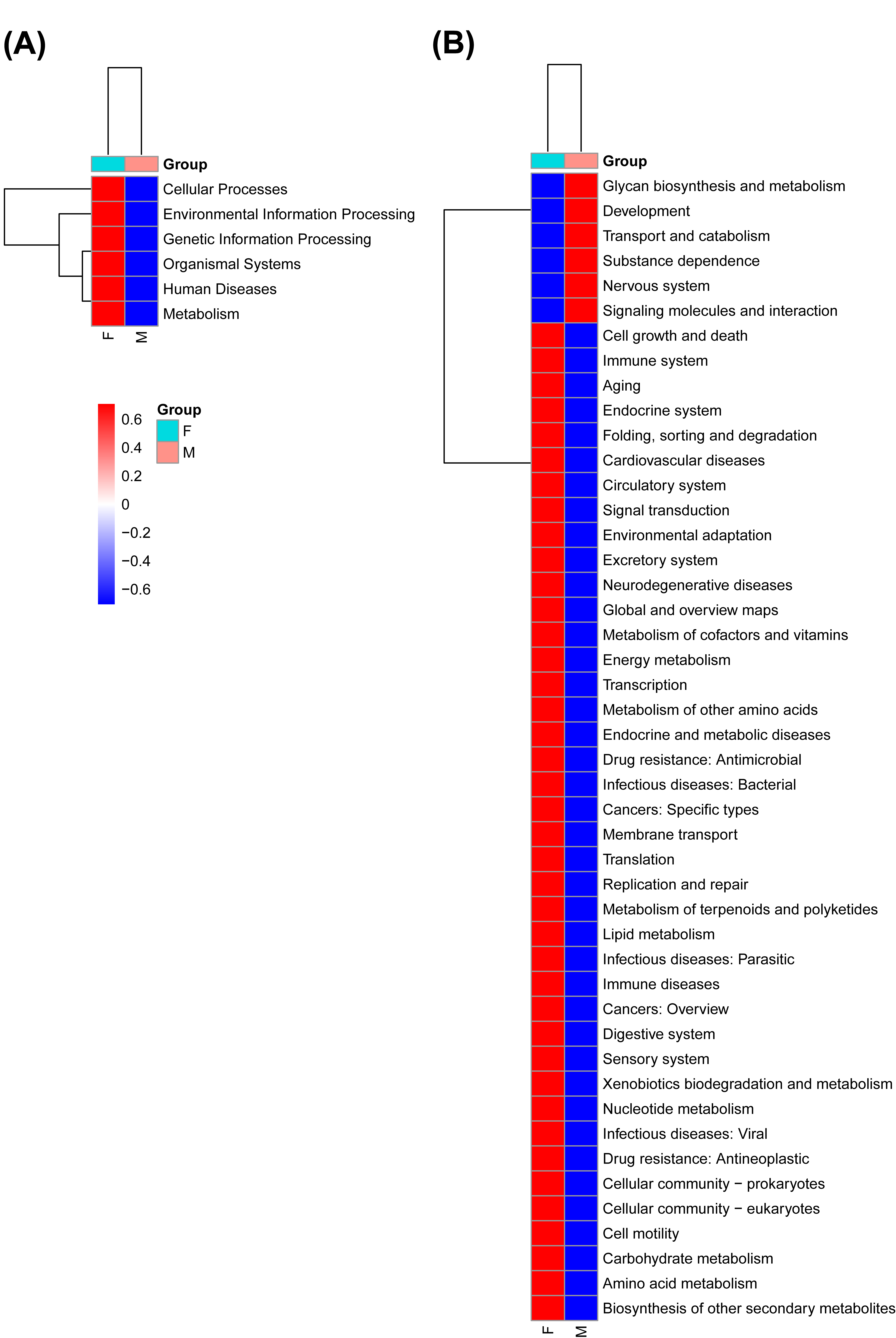
**
